# P05.59. Older adults’ attitudes on collaborative care of low back pain by doctors of chiropractic and medical doctors: a focus group study

**DOI:** 10.1186/1472-6882-12-S1-P419

**Published:** 2012-06-12

**Authors:** K Lyons, S Salsbury, M Hondras, M Jones, A Andresen, C Goertz

**Affiliations:** 1 Thomas Jefferson University, Philadelphia, USA; 2 Palmer Center for Chiropractic Research, Davenport, USA; 3 Genesis Quad Cities Family Medicine Residency Program, Davenport, USA

## Purpose

The purpose of this study was to assess the attitudes of older individuals with low back pain (LBP) toward collaboration between medical doctors (MD) and doctors of chiropractic (DC) for LBP treatment. The information obtained in these groups supported development of a collaborative care model based on an existing integrative medicine model (Hsiao et al, 2006).

## Methods

Ten focus groups were conducted with 48 unpaid volunteers, ranging in age from 65 to 90 who reported LBP in the past year. Participants were recruited from a family medicine clinic and chiropractic academic health center by invitational letter and through flyers posted at senior centers. Key questions discussed included participants’ treatment expectations, reasons for selecting an MD or DC as the primary LBP provider, extent to which care by alternate providers was discussed with their primary LBP provider, and attitudes toward a collaborative model of LBP care.

## Results

Overall, responses were consistent across groups. Participants’ treatment expectations included pain relief, the ability to remain active, to be treated as a person, and substantive treatment plan involvement. MDs were chosen for acute pain relief requiring medication, while DCs were chosen for addressing chronic pain or musculoskeletal defects. Participants reported little hesitancy to discuss treatment from alternate practitioners with their primary LBP provider and felt positively about LBP co-management by a DC-MD team. Most of these older adults were willing to participate in such a model of care if time, travel and cost concerns were addressed. Participants stressed the importance of real communication, including health record sharing, between the two providers and that each should have respect for the other.

## Conclusion

The results of these focus groups provided useful information in the development of the MD-DC co-management model and suggested significant interest on the part of older adults to participate in an alternative model of LBP care.

